# Keep distance and hear me out! Swedish-speaking children’s pandemic narratives informing rights-based approaches to future crises

**DOI:** 10.3389/fpubh.2025.1636066

**Published:** 2025-09-03

**Authors:** Mazen Baroudi, Shahd El Nabris, Malale Tungu, Hajime Takeuchi

**Affiliations:** ^1^Department of Epidemiology and Global Health, Umeå University, Umeå, Sweden; ^2^Department of Development Studies, School of Public Health and Social Sciences, Muhimbili University of Health and Allied Sciences, Dar es Salaam, Tanzania; ^3^School of Social Welfare, Bukkyo University, Kyoto, Japan

**Keywords:** children’s rights, COVID-19, Sweden, UNCRC, equity, participation, mental health, resilience

## Abstract

**Background:**

The COVID-19 pandemic disrupted children’s lives globally, yet limited research centres their voices in evaluating crisis responses. Sweden’s unique strategy, emphasizing voluntary guidelines rather than strict lockdowns, provides a critical context to explore how the pandemic policies reshaped children’s daily lives and affected systemic inequities between them; and to highlight children’s perspectives on how societal actors could better uphold children’s rights in future crises.

**Methods:**

This qualitative study engaged 44 Swedish-speaking children (aged 10–17) from diverse neighborhoods in Umeå, Sweden, through ten focus group discussions conducted between February 2023 and January 2024. Child-friendly adaptations of the United Nations Convention on the Rights of the Child (UNCRC) articles guided discussions on pandemic experiences. Data included transcripts, participant drawings, and field notes, analysed via reflexive thematic analysis.

**Results:**

The findings consolidate into four central themes. First, *“Keep distance! COVID forming a new way of life”* captures how Sweden’s voluntary measures reshaped daily norms, while prolonged isolation from quarantines, canceled activities, and restricted social interactions deepened confinement. Second, *“Erosion of well-being and need for information and support”* highlights the decline in physical health and mental well-being, compounded by inadequate mental health resources and schools’ dual role as social refuges and infection risks. Third, *“Paradox of ‘Normalcy’: halted education and systemic inequities”* reveals how Sweden’s open-school strategy masked halted learning, digital divides, and housing disparities shaping quarantine experiences. Finally, *“Hear me out! Engaging children in decision-making”* underscores children’s critiques of exclusion from policymaking, despite their resilience and proposals for participatory solutions.

**Conclusion:**

This study emphasizes the need to incorporate children’s voices in policymaking, particularly during crises, to ensure their rights and well-being are upheld. It calls for a shift in crisis response beyond just physical safety to include mental and emotional health, highlighting the importance of school-based mental health services and tailored support for marginalized groups in Sweden. To truly implement UNCRC Article 12, the country should establish formal systems that actively engage children and consider their feedback in decision-making processes.

## 1 Introduction

The measures implemented in response to the COVID-19 pandemic have disrupted the lives of children worldwide, with profound implications for their physical, emotional, and social well-being (1). While 60% of children globally lived in countries with total lockdowns impacting their education and social connections (2), pandemic mitigation strategies in Sweden diverged from global norms. Swedish authorities relied on voluntary measures such as social distancing and hygiene instructions (3). Secondary schools switched to online education for limited time periods while schools for children under 16 stayed open for in-person learning, with some restrictions on extracurricular activities. However, schools had the autonomy to opt for temporary shifts to online teaching depending on the individual school’s judgment (3, 4). While this approach preserved certain freedoms, its impact on children’s self-perceived rights, especially among marginalized groups such as immigrants, remains underexplored (5).

The United Nations Convention on the Rights of the Child (UNCRC), ratified by Sweden in 1990, is the most comprehensive international instrument on children’s rights (6). Sweden has been a progressive advocate for children’s rights. Legislative reforms have strengthened children’s rights, culminating in a 2020 bill fully integrating the UN-CRC into Swedish law (7). The UNCRC, emphasizes the importance of prioritizing children’s voices in policies affecting their lives especially under crisis such as the COVID-19 pandemic (8). However, pandemic responses in many countries, including Sweden, predominantly reflected adult perspectives sidelining children’s voices (9).

Research on children’s pandemic experiences in Sweden has focused largely on quantitative assessments of mental health or adults’ reports of child well-being. Worries and fears mainly about illness, death, and the future were common among Swedish children, with adolescents concerned about lost opportunities in youth experiences and employment (10). School nurses’ perspectives highlighted a declining mental health during the COVID-19 pandemic, particularly among girls and children in disadvantaged areas (11). Moreover, children’s viewpoints in Swedish media were selected and represented primarily by adults, resulting in limited inclusion overlooking children’s agency in articulating their rights (12). Qualitative insight into how children themselves understood and navigated rights-based challenges remain sparse. This omission is critical, as children’s interpretations of rights violations may differ from adult assumptions and children’s self-reported experiences of rights (13).

Through a qualitative, child-cantered lens, this study addresses the forementioned gaps by exploring Swedish-speaking children’s lived experiences of their rights under the UNCRC during the COVID-19 pandemic. The study aimed to explore how the pandemic policies reshaped children’s daily lives and affected systemic inequities between them; and to highlight children’s perspectives on how societal actors could better uphold children’s rights in future crises.

## 2 Methods

### 2.1 Study setting

The study was conducted in Umeå, a university city in northern Sweden (Västerbotten County) with a population of approximately 130,000 residents. Approximately 18% of Umeå’s population is foreign-born. Data collection occurred between February 2023 and January 2024; a period marked by Sweden’s transition to a post-pandemic “normalization” phase. However, the study focused on children’s reflections on their lives during the 2020–2022 COVID-19 pandemic, when Sweden adopted a unique mitigation strategy emphasizing voluntary guidelines rather than strict lockdowns.

### 2.2 Study design and sampling

This study adopted a qualitative exploratory design. The design prioritized participatory principles aligned with Article 12 of the UNCRC, emphasizing children’s agency as active narrators of their lived realities. Focus group discussions (FGDs) were selected as the primary method to leverage peer interactions, which can stimulate candid dialogue and reduce power imbalances between children and adult researchers. Participants included children aged 10–17 years residing in Umeå, Sweden, during the COVID-19 pandemic who speak Swedish fluently (to ensure comprehension of discussion materials). Exclusion criteria included inability to participate in group discussions due to cognitive or language barriers.

The participants were recruited through purposive sampling through collaboration with a local leisure activity organization. Recruitment materials were distributed via the local leisure activity organization multilingual staff. The partnership with this organization ensured culturally sensitive recruitment to engage families from immigrant backgrounds. It also ensured accessibility and familiarity for participants, fostering a comfortable environment for dialogue.

### 2.3 Participants and data collection

Ten focus group discussions (FGDs) were conducted each comprising 3–5 participants to balance dynamic interaction with individual voice. A total of 44 children participated (16 girls, 28 boys) who lived in diverse residential neighborhoods across Umeå and were born outside Sweden (19), in Sweden for parents born outside Sweden (13) or in Sweden for Swedish-born parents (12). The geographic and demographic diversity ensured representation of children from varying socio-economic backgrounds and living conditions, which informed their pandemic experiences (e.g., access to outdoor spaces, housing density). The children are aged 10 to 12 years (22 participants), 13 to 14 years (16 participants) and 15 to 17 years (6 participants). The FGDs included children of various age groups to foster a dynamic and inclusive discussion. Recognizing the potential power imbalances stemming from age differences and varying levels of confidence or talkativeness among participants, the data collection team included two trained youth moderators. Their presence was intended to reduce hierarchical dynamics often present between adult researchers and children. The moderators played a key role in creating a more equitable environment. As adult researchers, they actively reflected on their positions and took intentional steps to reduce perceived authority, such as using first names or nicknames and sharing personal pandemic experiences, to build rapport and encourage open dialogue. Sessions were also held in child-friendly, familiar spaces selected to minimize intimidation and evoke a sense of comfort and safety.

The sessions lasted 80–100 min, including one 20-min break with snacks to reduce fatigue. Each session was structured around two child-friendly adaptations of ten articles from the United Nations Convention on the Rights of the Child (UNCRC). These articles included the articles number 2 (no discrimination), 3 (best interests of the child), 6 (life, survival and development), 12 (respect for children’s views), 23 (children with disabilities), 24 (health, water, food and environment), 26 (social and economic help), 27 (food, clothing and safe home), 28 & 29 (access to and aims of education), and 31 (rest, play, culture and arts). The moderators introduced the Child-friendly UNCRC articles which were printed on posters as discussion prompts. Moderators used a standardized protocol with open-ended questions. The lead researcher observed all sessions to ensure consistency. Discrepancies (e.g., varying probe depth) were addressed in daily debriefs. Open-ended questions probed participants’ understanding of their rights, perceived impacts of the pandemic on these rights, and actionable measures to uphold them (e.g., “How did COVID-19 affect your ability to learn or play?”; “What could adults do better to protect children’s rights?”). Children were invited to draw experiences related to these rights. Drawings served as visual anchors to stimulate dialogue, encouraged quieter participants and the children to open up. However, many children dismissed the request to draw and offered written thoughts instead. All discussions were audio-recorded, transcribed verbatim, and enriched with field notes, moderator reflections, and participant-generated drawings.

### 2.4 Data analysis

Thematic analysis, following Braun and Clarke’s six-phase framework (14), was employed to identify, refine, and interpret patterns in the data. First, verbatim transcripts, participant drawings, and moderator notes were reviewed iteratively to ensure familiarity. All data sources were reviewed and coded collectively, allowing for the synthesis of recurring patterns into thematic clusters. Transcripts offered insight into how children interpreted their drawings, while field notes provided additional contextual depth which enriched the analytical understanding of both visual and verbal data. Initial manifesto codes were generated inductively through line-by-line analysis of all transcripts. Codes were then grouped into broader themes allowing interpretations and latent meaning using Word and Excel. The themes were refined through team discussions to ensure coherence and relevance to the research objectives. Transcripts were re-examined to ensure themes reflected the full dataset. Finally, each theme was clearly defined and illustrated with data excerpts drawn from different FGDs, reflecting the narrative and contextual richness of the data. Triangulation of audio recordings, notes, and visual data enhanced analytical rigor.

### 2.5 Strength and limitations

Using focus groups promotes dynamic interaction among participants, often eliciting insights that might not emerge in one-on-one interviews (15). The study’s diverse sample, including participants of varying genders, ages, and neighborhood contexts, helped ensure a broad representation of perspectives. However, conducting the discussions in Swedish may have inadvertently excluded children with limited language proficiency, potentially limiting the range of voices included. While data collection occurred post-pandemic, the study prioritized children’s meaning-making and interpretation of experiences, which remain valid and insightful in qualitative research despite the retrospective design.

Although the sample included children aged 10–17, age-stratified analysis was not conducted due to methodological and analytical limitations. Focus groups were intentionally mixed to foster inter-peer dialogue, which enriched discussion but prevented attributing responses to specific age brackets. Additionally, the uneven age distribution, particularly limited representation of older adolescents, hindered subgroup comparisons. Thematic overlaps across age groups further constrained stratification, as children shared similar rights-based concerns regardless of age. The study’s analytic focus remained on collective experiences in accordance to the participatory and rights-based design.

Incorporating child-friendly versions of the UNCRC articles into the discussions supported children’s comprehension of their rights, thereby enriching the quality of the data. Additionally, the moderator’s young age likely contributed to more effective communication and rapport with the participants. To enhance the credibility of the analysis, the research team actively reflected on their roles and assumptions throughout the study. Adult researchers worked to reduce hierarchical dynamics by sharing personal experiences and using informal communication, fostering openness and trust with participants. During analysis, themes were reviewed collaboratively to account for differing positional perspectives and ensure children’s voices remained central.

### 2.6 Ethical considerations

Ethical approval was obtained from the Swedish Ethical Review Board (Dnr 2022–04241-01). Written informed consent was secured from children 15 years old or older, while guardians of children under 15 provided written informed assent and their parents provided written or oral consent. Participants were assured of confidentiality, with data pseudonymized during transcription and stored securely on password-protected servers. Participants received gift vouchers (150 SEK) to acknowledge their time, distributed post-session to avoid coercion. The leisure organization facilitated voluntary participation, emphasizing children’s right to withdraw at any stage without consequence.

## 3 Results

**Figure 1:**
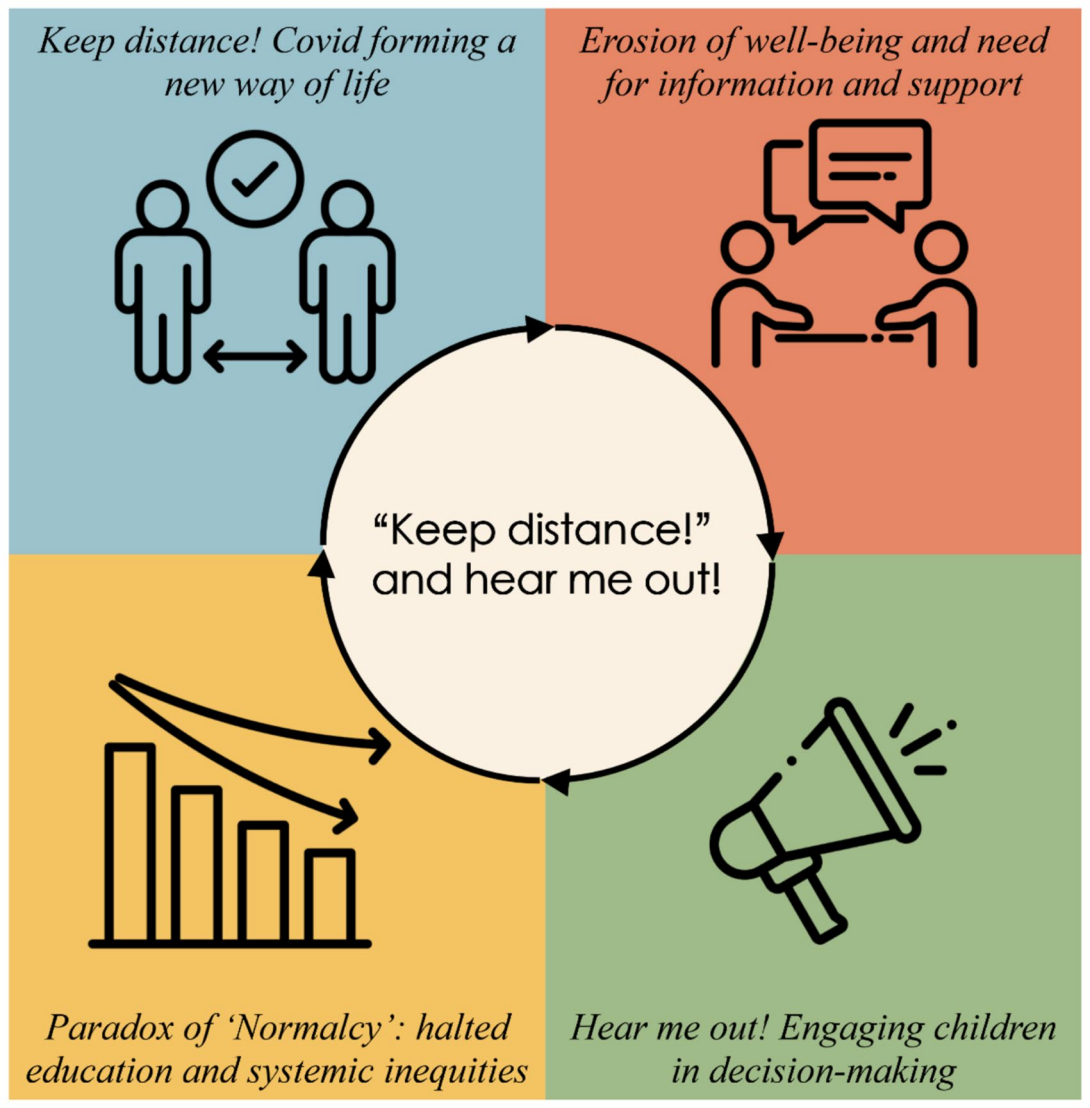
Swedish-speaking children’s experiences of their rights during the COVID-19 pandemic.

We developed four interconnected themes that explore Swedish-speaking children’s experiences of their rights during the COVID-19 pandemic (Figure 1). First, “*Keep distance! COVID forming a new way of life*” illustrates how Sweden’s voluntary safety measures, symbolized by the universal slogan “*Keep distance!*”, reshaped daily routines, embedding rules like handwashing and social distancing into everyday life. While children understood these measures, prolonged isolation from quarantines, canceled activities, and restricted social interactions created a pervasive sense of confinement. Second, “*Erosion of well-being and need for information and support*” underscores the pandemic’s toll on physical and mental health, exacerbated by inadequate mental health resources and school’s dual role as both a social refuge and infection risk. Third, the “*Paradox of ‘Normalcy’: halted education and systemic inequities*” exposes how Sweden’s open-school strategy masked halted learning, digital divides, and housing-based disparities, with remote learning and quarantines disproportionately affecting marginalized groups. Finally, “*Hear me out! Engaging children in decision-making*” highlights children’s critiques of their exclusion from policy discussions, despite their resilience and proposals for inclusive governance. Together, these themes reflect tensions between Sweden’s child-rights commitments and the realities of crisis policymaking.

### 3.1 Keep distance! COVID-19 is forming a new way of life

Children navigated Sweden’s unique pandemic strategy introducing rules that became embedded in daily routines. Participants recalled directives like social distancing and handwashing routines. The voluntary safety recommendations became a part of people’s everyday life. The prompt, “Keep distance!” was presented almost as a slogan for the pandemic. While they understood the rationale for these measures, the cumulative effect of restrictions such as quarantines for minor symptoms, canceled holidays, canceled leisure and sport activities and reduced social interactions, led to extended periods of isolation and reshaped daily life in ways that felt isolating and confining.

While most places remained accessible, many usual activities disappeared, leaving children with fewer options. Their free time suffered most, with reduced sports and extracurriculars affecting both recreation and development. COVID restrictions led to children spending more time at home, missing sports due to sick coaches and cancelations and the overall physical activities decreased. With the limited options, screen time surged. Instead of meeting up with friends after school and finding something fun to do, they instead went straight home, and socializing shifted to calls and social media apps. Some enjoyed extra screen time and even made new friends through online gaming. While this filled time, it affected physical activities and created a whole lot of boredom.

*(Child 2, FGD 5): “The problem was that if you just got a little cold then you had to stay home and do a corona test and then you had to wait like 2 days…”*

*(Child 3, FGD 5): “Well, the coach was ill, so the training was cancelled.”*

*(Child 4, FGD 5): “I was just at home… It was really boring seriously… I used to do facetiming with my friends but it was just only that.”*

*(Child 1, FGD 9): “It was mostly games, eyes on the screen. You sat and played, sort of. You played and ate, played and ate.”*

Most memories from the pandemic were of limitations including restrictions on public places such as malls, restaurants, and theatres. To adapt, people relied on delivery services or pre-ordering essentials for contactless pickup of clothes, food, and groceries. Yet, the pandemic created an opportunity for more times with family and for community solidarity where families supported quarantined neighbors.

*(Child 2, FGD 5): “Yeah… and you do not have to go into the store and get the stuff… They’ve already packed it and left a bag outside and then you just go and get it.”*

*(Child 1, FGD 5): “We had some neighbours… we went shopping for them and picked up the goods for them. That’s how we solved it and when we got sick, they did the opposite.”*

### 3.2 Erosion of well-being and need for information and support

The pandemic eroded children’s health and well-being. Restrictions reduced physical activity. Sports cancelations left children feeling “weaker,” though some cycled or exercised outdoors. Mental health suffered as isolation deepened with missing out on closeness and social interactions. Schools turned out to be the last resort of social interaction and a double-edged space: a refuge from isolation but also a site of infection risk. Concern for vulnerable relatives, like grandparents, was widespread. Some took precautions such as wearing gloves on buses and avoiding shared spaces while others dismissed the risks. This divide caused friction. Anxious children expressed frustration toward peers who ignored rules, revealing a divide in how safety measures were perceived and followed. Children also missed discussions about the Pandemic in schools and criticized the inaccessibility of mental health support services, such as school counselors. This left children to internalize fears about the risk of infection and vulnerable relatives.

Many noted feelings of being less healthy or weaker and that the body did not get what it needed. The children themselves emphasized the need to stay active and get fresh air. With limited social interactions, school became the only place to connect. However, children faced unavoidable infection risks, especially in schools. Despite measures like handwashing and keeping distance, many felt unprotected and took extra precautions, such as avoiding touching doors, wearing gloves, and airing out classrooms. Public spaces like transport and laundry rooms also posed risks, making some highly cautious. Concerns about “bad air” were common, with children feeling restricted indoors. Quarantine intensified these feelings, making the experience of being confined even more frustrating and heightening the emotional strain of the pandemic.

*(Child 2, FGD 10): “If you get coronavirus, you never go out, you breathe the air, and everything will become a coronavirus house.”*

*Leader: What do you mean?*

*(Child 2, FGD 10): “Well, if the whole house has corona and you never go out. Then it will be, maybe if you breathe in, then you will suck in all oxygen and the house become all carbon dioxide instead, so you cannot breathe.”*

Children had differing views on COVID-19. Some felt anxious, fearing for their health and that of loved ones, diligently following safety measures with an instilled sense of responsibility. Others saw it as just an illness, not a serious threat. This divide led some to defy restrictions out of indifference, unwillingness, or forgetfulness. Social distancing was especially difficult at school, and some neglected handwashing. Beliefs that COVID mainly affected older adults contributed to this. Regardless of personal fear of COVID-19, children shared concerns for family members, especially those in risk groups. Some had sick or hospitalized relatives and visiting them was difficult due to restrictions. Not being able to say final goodbyes deepened their sorrow, and they also witnessed their parents’ pain.

*(Child 4, FGD 7): “The infection spreads through the school, but no one cared, everyone was like half a centimetre away from each other.”*

*(Child 1, FGD 4): “Yes, many in our class or not many but some in our class like “eh who cares” … And then you got a little angry because they do not understand the seriousness of it sometimes… You feel a little like this, should others suffer because you cannot wash your hands… The first thought when I heard about Corona was that my grandparents will die.”*

*(Child 1, FGD 5): “It was only scary for me because of my grandparents… For me I do not care if I get corona.”*

*(Child 3, FGD 9): “My grandmother was so sick, and we were not allowed to see her in the hospital before she died.”*

Children sought both support and more information about COVID-19, expressing frustration over insufficient conversation about the virus. Schools provided some information, often through Lilla Aktuellt (a news program directed to children), but many children wanted more dialogue, especially to address circulating rumors and help achieve higher degree of compliance by those who defied restrictions. Some felt media coverage, particularly news, exaggerated the situation, fuelling fears. They stressed the need for better rational and calming information from media and teachers, as educators played a crucial role in shaping students’ attitudes and behaviors. Despite a straining period for most children with fears, worries, unanswered questions and stress being higher than ever, participants were lacking a place to turn to for support. Schools’ nurses and counselors were often unavailable, leaving some to cope alone. Many hesitated to seek help, wishing for support that reached out to them instead. They desired more accessible, COVID-specific mental health resources to guide them.

*(Child 1, FGD 4): “We only saw ‘Lilla Aktuellt’ at school on Fridays and that was the only thing they told us about corona. You could have had a talk with the whole class about Corona and people who had questions about it, but this never really happened in our class. Rumours spread about Corona and then you could be even more afraid.”*

*(Child 4, FGD 1): “Yes, the teachers did not talk much about it like they kind of did not care.”*

*(Child 1, FGD 4): “I was very often afraid of my grandparents, but I did not dare to talk about it with dad and at school they did not offer a psychologist, or you could not talk to the school nurse… she was just there but she did not help anyone.”*

*(Child 3, FGD 4):” Well, ours used to be at school only on Mondays… And then she was always busy.”*

*(Child 1, FGD 4): “…it was more that I just went and kept it in my head until the pandemic was over.”*

### 3.3 Paradox of “Normalcy”: halted education and systemic inequities

The Swedish strategy of voluntary safety recommendation and open schools masked halted education and led to inequal experiences. Some classes shifted online; others stayed open with distancing. Even when teaching was in place, the learning halted due to absences because of illness and quarantines. Remote learning exposed digital divides where some schools provided laptops to their students while others did not, leaving some children dependent on phone to attend online lessons. Additionally, quarantining experiences varied significantly depending on the home environment, such as crowded apartments versus houses with own yards.

During the pandemic, students and teachers faced significant challenges that affected the quality and time spent on education. Absenteeism due to illness was a major factor, with many students missing school for extended periods, and sometimes entire schools would close temporarily due to infections. The quality of education was also impacted, especially when students had substitute teachers who were not familiar with the subject matter. This lack of continuity made learning less effective. With disruptions of the education process, many students struggled to keep up with their studies. Teachers had to slow down the pace to accommodate those who had fallen behind, making lessons feel less productive. As a result, many students felt they were not where they should be in terms of learning, and after the pandemic, gaps in their knowledge became more apparent.

*(Child 3, FGD 3): “If you had a stuffy nose or cough, they sent you home right away… even if you did not feel sick.”*

*(Child 2, FGD 5): “… you want to be at home but you are going to miss a lot so when you go back it’s going to be quite hard.”*

*(Child 1, FGD 4): “So, they did not want us to go too far ahead because then 70% of the class would have to catch up… It did not feel like real lessons… You kind of had to back up.”*

*(Child 3, FGD 4): “Or stop.”*

*(Child 1, FGD 5): “We have to repeat things that we learned … and no one could cope so it was really hard, I think it was harder when corona was over than when it was in…”*

While many children were unsure about preferring in-school or online education, some saw advantages in remote learning. Distance learning made students feel safer, especially during high infection periods, as it reduced the risk of exposure. It also allowed for more interaction with others, unlike restricted in-school education with distancing measures. Some found remote education more accessible and less disruptive, offering a calm environment for better concentration. Also, students attending school sometimes felt it was like remote learning, with independent work on computers or video calls with teachers, leading to frustration over a lack of understanding and insufficient help during lessons.

*(Child 2, FGD 7): “To be honest, it feels like it was almost a little better [distance learning] … you were on your own, so it was quieter and easier to concentrate.”*

*(Child 3, FGD 7): Yes, I thought it was good because you feel safer and less chance of getting infected and you got to spend time with other people instead of sitting on a chair and working all the time.”*

*(Child 3, FGD 3): “Well, you were not allowed to sit so close to your friends, it was harder to ask for help and then you had to ask the teacher, and the teacher ran around with a mask, so it was really hard to talk or get help.”*

*(Child 2, FGD 5): “If you do Zoom, everyone can come even if sick, but if you got sick and the teaching is in place then you will not be able to come.”*

However, many children preferred in-person education during the pandemic because it allowed them to socialize with friends, something online learning could not provide. Remote learning also presented numerous distractions, like notifications and the temptation to rest or play at home, which made it harder to focus. Without a teacher physically present to manage the class, some students struggled to stay engaged, and classes became chaotic. The lack of immediate help from teachers also hindered learning, as students found it difficult to get assistance with assignments. This was particularly challenging for students requiring additional support, such as newly immigrated children or those with learning disabilities. Transitioning to remote learning posed challenges for both students and teachers, leading to lower engagement and less productive learning. Many participants felt that the setback caused by remote learning would have long-term effects on their education.

*(Child 3, FGD 3): “… when you are at school, the teachers are there and you have to work, but if you were at home, you might look at your mobile phone, eat something, walk around, talk to others… (FGD 3a)*

*(Child 4, FGD 3): “Yeah, our class was messy because everyone saw the chance to … well ‘I do not have to listen … what can they do!’.”*

The uncertainty surrounding COVID-19 led to confusion, especially regarding school decisions. Schools remained open but could switch to distance learning as needed. The lack of clear direction resulted in inequal experiences, with some schools fully implementing remote learning, others trying it intermittently, and some not at all. This caused frustration and disappointment among students about having or not having the teaching in a certain way, especially since the differences were between schools in the same town or between different classes at the same school. Many felt disappointed by inconsistent rules, particularly when schools adjusted based on infection rates.

*(Child 1, FGD 7): “… why did not we get distance, they said if many get corona, then we get distance, it was almost the whole school that got corona.”*

Another inequal experience arose from access to electronic devices; those with personal devices such as phones, tablets, or video games had better experience of free time during the pandemic. Distance learning also exposed further disparities. Some schools provided laptops to their students, while others did not, hindering equal access to education. Additionally, the home environment played a significant role in how children experienced quarantine. Some had spacious homes with access to outdoor areas, while others living in crowded environments faced limitations.

*(Child 3, FGD 5): “We live in a single-family house so I could go out to the backyard if I needed air. And we have a trampoline so I could move around quite well.”*

### 3.4 Hear me out! engaging children in decision making

While most children largely praised Sweden’s strategy of no strict luck down, some felt that children’s rights were neglected, and their voices were not included in decisions. Many children felt excluded from the conversation and wanted more opportunities to express their opinions. While some believed that adults should make the decisions, others felt that both children and adults should be involved in decision-making, emphasizing the importance of considering children’s perspectives during the pandemic.

During the pandemic, most children were satisfied with how the government handled the crisis claiming that “they did the best they could” and believing that Sweden’s strategy allowed for a more normal life compared to other countries. The widespread distribution of safety recommendations, including posters about hand hygiene and social distancing, and the easy access to masks and hand sanitizers were particularly appreciated. Many appreciated also the convenience and safety of receiving vaccinations at school, where they felt supported by friends or trusted adults.

*(Child 3, FGD 2): “We had the best of all the other, we lived like a normal life compared to the others. We could go out, just that we should keep our distance, and we had less infection rate than other countries. And the health of the children was better because the children were allowed to do what they wanted.”*

Even though participants were mostly pleased with the policies of the government they felt that the government was too busy during pandemic and therefore did not have time to think about the rights of children. Even more, the participants felt that they were not included in the conversation and that their opinions were not heard. When the participants were asked about how things could improve or which changes they wanted enforced, school remained a central part of children’s lives, and most of their suggestions for improvement focused on this area. As mentioned in a previous theme, they highlighted the need for more classroom discussions and information and increased the availability of school counselors. They additionally suggested a website where children could turn to for psychological support. to reduce the burden and improve the overall experience during the pandemic, the children suggested increasing outdoor activities, starting more youth centres, and lowering sports costs.

Participants also emphasized the importance of children’s voices being heard, suggesting school council meetings, voting, or apps that children can use to reach policy makers anonymously. They proposed sending governmental surveys or using social media to gather opinions.

*(Child 3, FGD 5): “… well it felt more like they were focusing on making old people less likely to get infected and then we, the children are affected.”*

*(Child 4, FGD 3): “Yeah, they should ask the children about their rights and how they feel. Yes, it can change a lot if you try to do something about it, fix it so that it gets better.”*

*(Child 2, FGD 6): “We have school councils, and it feels like we can make a difference because when we give advice to the teachers, they think about it and try to improve the school.”*

## 4 Discussion

Even though Sweden had a unique and highly debated strategy which allowed for the society to keep open, it did not completely hinder the effects of the pandemic which were felt by the children in their free time and day-to-day life. This study aimed to explore children’s rights during the COVID-19 pandemic in Sweden revealing critical tensions between Sweden’s lauded pandemic strategy and children’s lived realities. The results show that children’s perspectives and experiences during the pandemic are valuable for understanding the complex challenges they faced and how their rights can be protected and promoted in future crises (16).

Anxiety and depression were common among children worldwide where specific groups having more vulnerabilities to this burden (17). Studies indicated heightened anxiety in children related to illness, reflecting both the psychological impacts of the pandemic and the fear generated through media and social discussions about COVID-19 (18). Our study presented a range of emotions among children during the pandemic that was highlighted in previous studies, including fear of contracting the virus or losing loved ones, and stress due to following hygiene rules and instructions (10, 19–21).

The Swedish health care system faced major challenges with higher incidence and mortality rate compared to its neighboring countries at the start of the Pandemic, and increased workload stress levels among healthcare workers (22, 23). This influenced the availability of mental health support, particularly for children. School health services in Sweden aim to promote students’ well-being and educational success through preventive care and early intervention, as mandated by the Swedish Education Act (Skollag 2010:800). These services involve school doctors, nurses, psychologists, counselors, and special educators. School health teams should collaborate with educators, social services, and healthcare providers to foster supportive learning environments (24). However, a 2024 report highlights widespread recruitment challenges particularly in recruiting special educators, psychologists, and nurses leading municipalities to rely on contracted services and remote support. This has resulted in inconsistent service quality and unequal access, particularly in rural areas (25). Reports have emphasized the necessity for increased resources in schools to better support children’s mental well-being and facilitate necessary interventions during challenging times (20, 26). A crucial insight derived from this situation is the need to prioritize children’s mental health within crisis preparedness frameworks and ongoing school health care initiatives. Having an adequate number of school nurses and mental health professionals available is essential for dealing with physical and mental health needs during crises.

Although some primary schools in Sweden were closed for various periods due to high COVID-19 cases, Sweden is noted for keeping primary schools open for children under 16 years of age during the early phases of the pandemic, making it only OECD country to adopt such an approach (2, 27). Further restrictions on sports tournaments were introduced March 2021 (28). While Sweden managed to maintain certain aspects of children’s lives as relatively normal, this “normalcy” concealed systemic inequities exacerbated by the pandemic; students with economically disadvantaged backgrounds or learning disabilities were not given sufficient consideration. Previous research highlighted the varying capacities among families to support remote learning (29). Students dependent on phones for online education struggled to engage fully, underscoring the necessity for equal access to educational resources, such as technical equipment and reliable internet connectivity (30, 31). These disparities are also apparent by the experiences shared by children with disabilities or special educational needs. Studies indicate that these children faced significant challenges during the transition to remote learning, often lacking the necessary support and resources to adapt effectively (32). These insights underscore the need for equitable access to educational resources to mitigate the inequities exacerbated by the pandemic.

The COVID-19 pandemic highlighted communication as a critical factor in crisis management, particularly in relation to upholding children’s rights (33). Effective communication is not only about disseminating accurate and accessible information but also about establishing mechanisms that genuinely include children in dialogue. Scholars emphasize the need for two-way communication channels that actively prioritize children’s voices, enabling them to express their concerns, experiences, and needs (34). This goes beyond simply “giving” children a voice; it requires creating structured, ongoing opportunities for children to participate meaningfully in decisions that affect them. During the pandemic, a lack of child-focused communication strategies exposed gaps in how decision-makers interact with young people, particularly regarding transparency, inclusivity, and responsiveness. These gaps underscore the need for institutionalized approaches, such as participatory mechanisms embedded in schools or local governance structures, that ensure children’s perspectives are not only heard but also acted upon (35). Addressing these issues is essential to advancing the holistic well-being of children and operationalizing Article 12 of the UNCRC in practice.

## 5 Conclusion

Overall, this study underlines the importance of integrating children’s perspectives in the design and implementation of policies and actions, especially during crises. By drawing on children’s experiences and suggestions, we can create a more inclusive and equitable future where children’s rights are protected and promoted, even in the most challenging circumstances. Crisis policies must move beyond physical safety to uphold holistic well-being.

Sweden must prioritize expanding school-based mental health services, and targeted support for marginalized groups. To operationalize UNCRC Article 12, Sweden could institutionalize participatory mechanisms to communicate and get feedback from children.

## Data Availability

The datasets presented in this article are not readily available because ethical and legal restrictions. Requests to access the datasets should be directed to Etikprövningsmyndigheten, Box 2,110, 750 02 Uppsala, registrator@etikprovning.se.
